# Myocardin and Stat3 act synergistically to inhibit cardiomyocyte apoptosis

**DOI:** 10.18632/oncotarget.20450

**Published:** 2017-08-24

**Authors:** Yuan Xiang, Xing-Hua Liao, Jia-Peng Li, Hui Li, Huan Qin, Ao Yao, Cheng-Xi Yu, Peng Hu, Wei Guo, Chao-Jiang Gu, Tong-Cun Zhang

**Affiliations:** ^1^ Institute of Biology and Medicine, Wuhan University of Science and Technology, Hubei, 430081, P.R. China; ^2^ Key Laboratory of Industrial Fermentation Microbiology, Ministry of Education and Tianjin, College of Biotechnology, Tianjin University of Science and Technology, Tianjin, 300457, P.R. China; ^3^ Shenzhen Ritzcon Biological Technology Co., LTD, Shenzhen, Guangdong, 518000, P.R. China

**Keywords:** myocardial, Stat3, cardiomyocyte apoptosis

## Abstract

Signal transducer and activator of transcription 3 (Stat3) and Myocardin regulate cardiomyocyte differentiation, proliferation, and apoptosis. We report a novel aspect of the cellular function of Myocardin and Stat3 in the regulation of cardiomyocyte apoptosis. Myocardin and Stat3 showed anti-apoptotic function by increasing the expression of Bcl-2 while reducing expression of the pro-apoptotic genes Bax, Apaf-1, caspase-9, and caspase-3. Moreover, myocardin/Stat3-mediated activation of Bcl-2 and Mcl-1 transcription is contingent on the CArG box. Myocardin and Stat3 synergistically inhibited staurosporine-induced cardiomyocyte apoptosis by up-regulating expression of anti-apoptotic Bcl-2 and Mcl-1 in neonatal rat cardiomyocytes. These results describe a novel anti-apoptotic Myocardin/Stat3 signaling pathway operating during cardiomyocyte apoptosis. This provides a molecular explanation for cardiomyocyte apoptosis inhibition as a critical component of myocardial protection.

## INTRODUCTION

Cardiomyocyte apoptosis is a key turning point in the cell death process [1–3], and critical in the pathogenesis of various cardiovascular diseases. Apoptosis inhibition is an important target for therapeutic intervention. However, the mechanism of cardiomyocyte apoptosis is not well understood.

Signal transducer and activator of transcription 3 (Stat3) is a kind of transcription which mediated cell growth, differentiation, and survival of various cell types [4–7]. Stat3 activation results in the upregulation of various genes involved in cell survival and proliferation, such as those encoding Bcl-2, Bcl-XL, Mcl-1 (myeloid cell leukemia-1), cyclin-D1 and c-Myc [8–10]. Inhibition of Stat3 activity reduced the expression of survivin in primary effusion lymphoma-induced cell apoptosis [11]. Importantly, constitutive cardiomyocyte- restricted deletion of Stat3 results in increased apoptosis [12, 13]. In this study, we demonstrate that Stat3 and Myocardin directly interact *in vivo*, suggesting that these two transcription factors synergistically enhance the anti-apoptosis effect in cardiomyocytes.

Myocardin is highly expressed in embryonic cardiac and smooth muscle [14–16], acting as a potent transcriptional activator that activates CArG box-dependent cardiac promoters by forming a ternary complex with SRF [17–19]. Previous studies have shown that SRF protects cells from apoptotic cell death by regulating anti-apoptosis gene Bcl-2 and Mcl-1 activity [20, 21]. Myocardin inhibits the cell-cycle progression at the G2/M phase [22], influences failing heart gene expression and function [23, 24], and Myocardin loss in cardiomyocytes triggers programmed cell death [25]. However, the molecular mechanism of Myocardin in cardiomyocyte apoptosis mediated-heart failure remains unknown.

We hypothesized that Myocardin and Stat3 have a synergistic anti-apoptosis effect in cardiomyocytes. Herein we demonstrate an interaction between Myocardin and Stat3 to inhibit apoptosis and upregulate anti-apoptotic gene expression for myocardial protection through cardiomyocyte apoptosis. We define a new role for Myocardin and Stat3 as a transcriptional repressor and inhibitor of cardiomyocyte apoptosis.

## RESULTS

### Myocardin and Stat3 synergistically inhibit staurosporine-induced cardiomyocyte apoptosis

**Figure 1:**
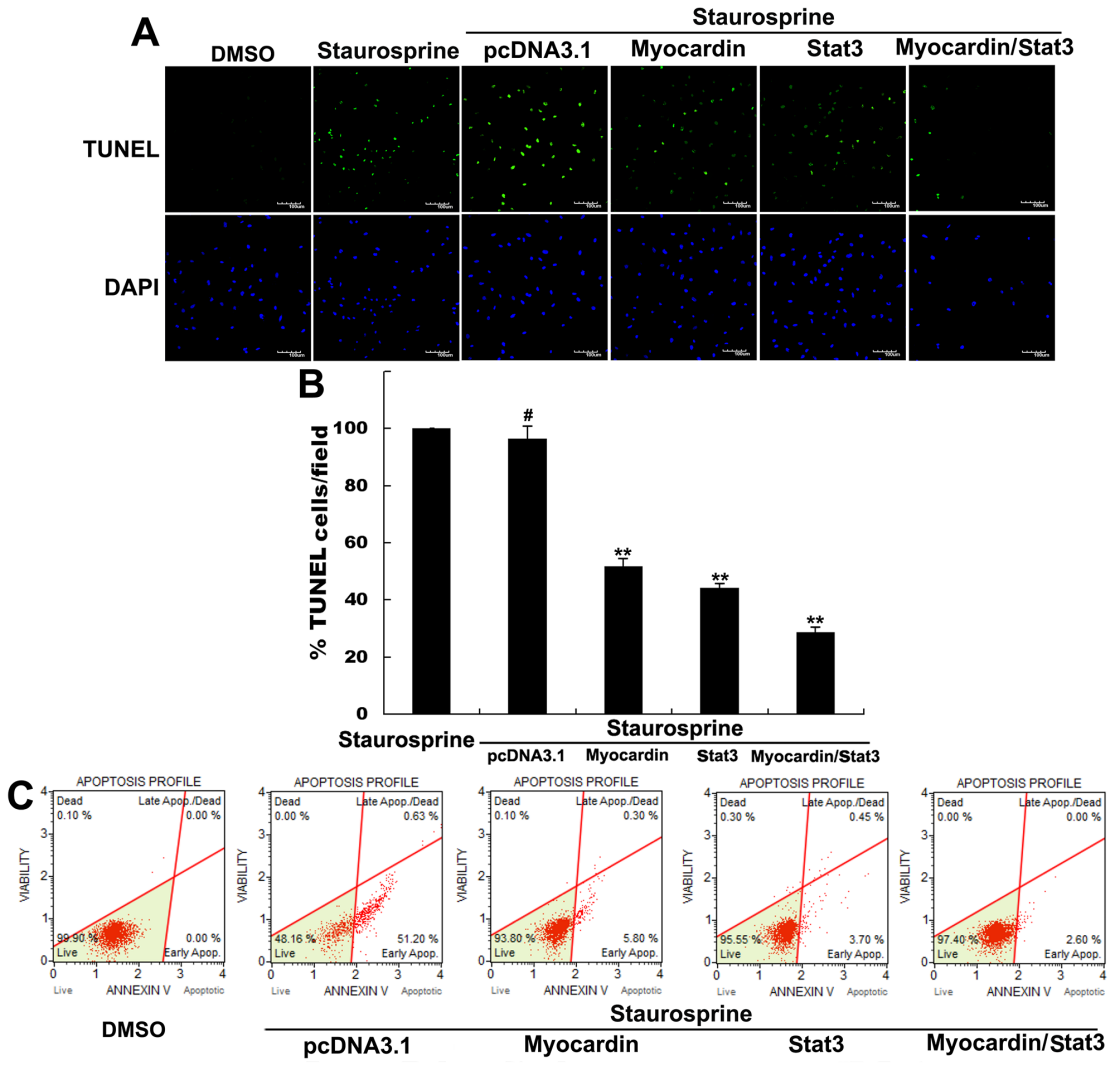
Myocardin and Stat3 inhibit staurosporine-induced cardiomyocyte apoptosis **(A)** Afterstimulating postnatal rat cardiomyocytes with 1μM staurosporine for 30 min to induce cardiomyocyte apoptosis, cells were transfected with the plasmids pcDNA3.1, Myocardin, Stat3, and Myocardin/Stat3 for 24 hours. Then we used TUNEL Assay to observe the change of apoptotic rate from treatment with staurosporine or transfection with the plasmids pcDNA3.1, Myocardin, Stat3, and Myocardin/Stat3 in the presence of staurosporine. **(B)** Statistical software was used to analyze the change in apoptotic rate by the TUNEL Assay. ^**^, *p*<0.01, ^#^, *p*>0.05. n=3. **(C)** Afterstimulating postnatal rat cardiomyocytes with 1μM staurosporine for 30min induce cardiomyocyte apoptosis, cells were transfected with the plasmids pcDNA3.1, Myocardin, Stat3, and Myocardin/Stat3 for 24 hours. Annexin V-FITC apoptosis detection assay was used to observe the change of apoptotic rate by treatment with staurosporine or transfection with the plasmids pcDNA3.1, Myocardin, Stat3, and Myocardin/Stat3 in the presence of staurosporine.

Both myocardin and Stat3 can mediate cardiomyocyte growth, differentiation, and survival, so we determined the role of Myocardin and Stat3 in cardiomyocyte apoptosis. The TUNEL assay results for Myocardin transfected cardiomyocyte cells indicated a decrease of nearly 50% in TUNEL-positive cells in staurosporine-treated cells as compared to the controls, while Stat3 transfected cardiomyocyte cells indicated a decrease of almost 55% (Figure 1A and 1B). Moreover, Myocardin and Stat3 cotransfected cardiomyocyte cells indicated an approximately 70% decrease (Figure 1A and 1B). Very few TUNEL-positive cells were present in the control cells. Importantly, the Annexin V-FITC apoptosis detection assay data quantify early and late events in the course of apoptosis. The Annexin V-FITC results show that Myocardin significantly diminished the number of apoptotic cells induced by staurosporine (Figure 1C). Similarly, Stat3 significantly reduced the apoptosis rate (Figure 1C). The observed number of apoptotic cells cotransfected with Myocardin and Stat3 declined (Figure 1C). These data suggest that cooperativity of Myocardin and Stat3 inhibits staurosporine-induced cardiomyocyte apoptosis.

### Myocardin and Stat3 enhance the expression of anti-apoptotic genes

**Figure 2:**
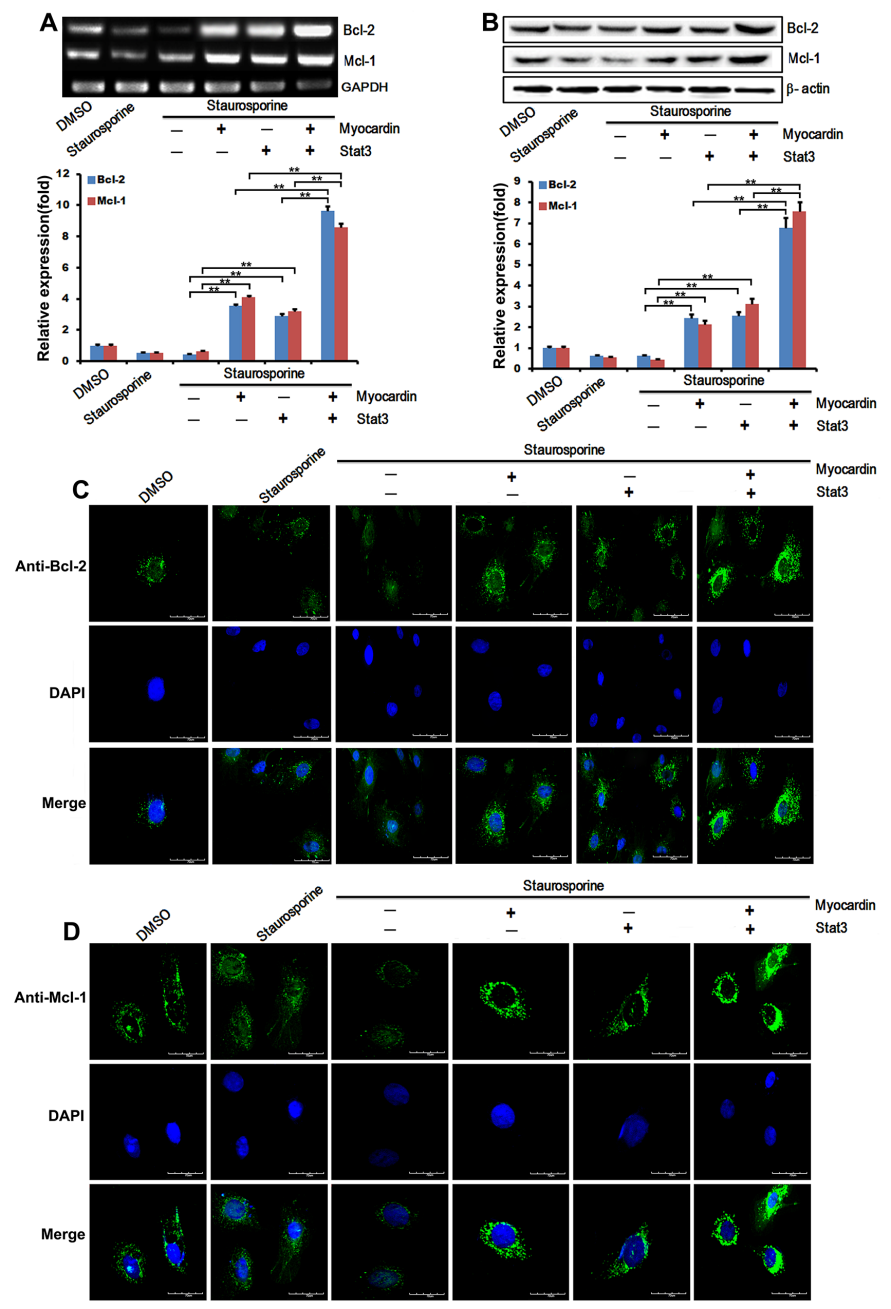
Myocardin and Stat3 enhance the expression of anti-apoptotic genes **(A)** Afterstimulating postnatal rat cardiomyocytes with 1μM staurosporine for 30min induce cardiomyocyte apoptosis, cells were transfected with the plasmids pcDNA3.1, Myocardin, Stat3, and Myocardin/Stat3 for 24 hours. Total RNAs were isolated, and the expression of Bcl-2 and Mcl-1 was examined by RT-PCR (including quantifications performed with Quantity One software). ^**^, *p*<0.01. n=3. **(B)** Afterstimulating postnatal rat cardiomyocytes with 1μM staurosporine for 30min induce cardiomyocyte apoptosis, cells were transfected with the plasmids pcDNA3.1, Myocardin, Stat3, and Myocardin/Stat3 for 24 hours. Total proteins were extracted, and the protein levels of Bcl-2 and Mcl-1 were detected by western blot analysis (including quantifications performed with Quantity One software). β-actin was used as a loading control. ^**^, *p*<0.01. n=3. **(C** and **D)** Representative images of immunostained cardiomyocytes. The left panels (green) respectively show anti- Bcl-2 and Mcl-1 antibody reactivity to demonstrate gross morphology. The middle panels (blue) show the DAPI staining for nuclei. The right panels respectively show double immunostaining for Bcl-2 and Mcl-1 and nuclei. Scale = 70μm.

The expression of anti-apoptotic markers was examined by RT-PCR and western blot to demonstrate the effect of Myocardin and Stat3 on regulating the expression of the anti-apoptotic markers (Bcl-2 and Mcl-1) in the presence of staurosporine. Our data showed that both Myocardin and Stat3 mitigate the effect of staurosporine on the expression of anti-apoptotic genes. The expression of Bcl-2 and Mcl-1 was enhanced after transfection with Myocardin and dramatically upregulated when transfected with the two proteins (Figure 2A). Further expression changes were also measured on the protein level by western blotting, and enhanced expression of Bcl-2 and Mcl-1 was observed when transfected with Myocardin and Stat3 (Figure 2B). As shown in Figure 2C and 2D, Myocardin and Stat3 strongly amplified the expression of Bcl-2 and Mcl-1. Bcl-2 and Mcl-1 protein expression was increased in cardiomyocyte cells transfected with Myocardin, Stat3, and Myocardin/Stat3 compared to the cells transfected with the pcDNA3.1 vector by immunofluorescence staining. Notably, Bcl-2 and Mcl-1 were located in the cytoplasm when transfected with vector but accumulated around the nuclei in cells cotransfected with Myocardin and Stat3. These observations suggest that Myocardin and Stat3 synergistically enhance the expression of anti-apoptotic genes to inhibit staurosporine-induced cardiomyocyte apoptosis. Our results indicate that Myocardin and Stat3 play a pivotal role as an anti-apoptotic factor in cardiomyocyte cells, and targeting Myocardin and Stat3 activity led to the induction of anti-apoptosis activity.

### Myocardin and Stat3 mediated anti-apoptosis by the intrinsic or mitochondrial apoptotic pathway

**Figure 3:**
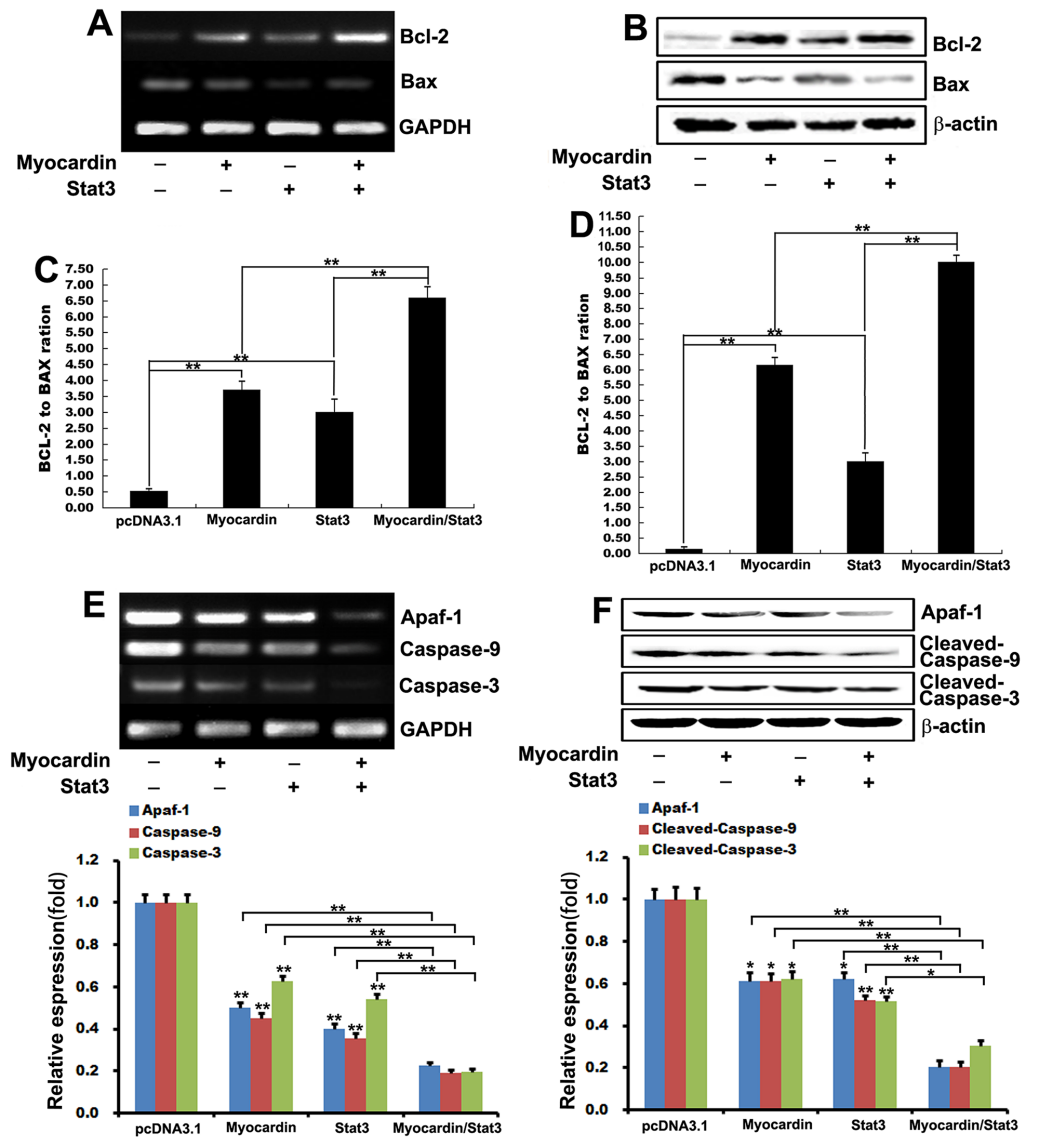
Myocardin and Stat3 mediated anti-apoptosis by the intrinsic or mitochondrial apoptotic pathway **(A and B)** The expression of Bcl-2 and Bax was examined by RT-PCR and western blot analysis. GAPDH and β-actin were used as loading controls. **(C and D)** The ratio of Bcl-2 to Bax was determined by statistical software. ^**^, *p*<0.01. n=3. **(E and F)** After postnatal rat cardiomyocytes were transfected with pcDNA3.1, Myocardin, Stat3, and Myocardin/Stat3 for 24 hours, the expression of Apaf-1, caspase-9 and caspase-3 were examined by RT-PCR and western blot analysis (including quantifications performed with Quantity One software). ^**^, *p*<0.01, ^*^, *p*<0.05. n=3.

It has been reported that the role of Bcl-2 family of proteins was evaluated by the measurement of the anti-apoptotic protein Bcl-2 and the pro-apoptotic protein Bax [26–29]. The expression of Bcl-2 was increased in Myocardin and Stat3 transfected cardiomyocytes (Figure 3A and 3B). Bax expression was decreased in the transfected Myocardin and Stat3 cardiomyocytes compared to the control cardiomyocytes (Figure 3A and 3B).

The functional significance of these expression patterns is derived from the potential for heterodimer formation between Bax and Bcl-2. A high Bax:Bcl-2 ratio enhances the probability for Bax homodimer formation and cell death signaling, whereas a relative abundance of Bcl-2 favors formation of Bcl-2/Bax heterodimers and Bcl-2/Bcl-2 homodimers, which promote cell survival [30, 31]. We investigated the mechanism of Myocardin and Stat3 enhancement of anti-apoptotic gene expression. We calculated the ratio of Bcl-2 expression to Bax expression, and the ratio was reversed in cardiomyocytes (elevated Bcl-2 levels and transient reduction of Bax levels). The ratio of Bcl-2:Bax indicated that when the ratio was more than 0.5, an apoptotic procedure switches on. The densitometric analysis showed a high Bcl-2/Bax ratio in transfected Myocardin and Stat3 cardiomyocytes (Figure 3C and 3D), which suggests that Myocardin and Stat3 have an anti-apoptotic function by increasing the expression of Bcl-2 while reducing the pro-apoptotic gene-Bax.

Because a low Bcl-2/Bax ratio enhances cell death signaling, we next determined apoptotic protease activating factor-1 (Apaf-1), the initiator caspase-9, and the effector caspase-3 expression. The cleavage of caspases is aided by the release of caspase-activating factors, particularly cytochrome C from the mitochondrial membrane into the cytosol [32, 33]. Once released into the cytosol, cytochrome C activates pro-caspase 9 [34]. Caspase-3 is considered the most important of the executioner caspases and is activated by the initiator caspase-9. Our results show that transfected Myocardin and Stat3 cardiomyocytes significantly reduce the release of cytochrome C and the expression of Apaf-1, caspase-9, and caspase-3 as compared to the controls (Figure 3E and 3F). These observations indicate that both Myocardin and Stat3 act as a potent anti-apoptotic by changing the ratio of Bcl-2/Bax, which reduces the release of cytochrome C and the expression of Apaf-1, caspase-9, and caspase-3 to affect the intrinsic or mitochondrial apoptotic pathway.

### Myocardin and Stat3 directly interact *in vivo*

**Figure 4:**
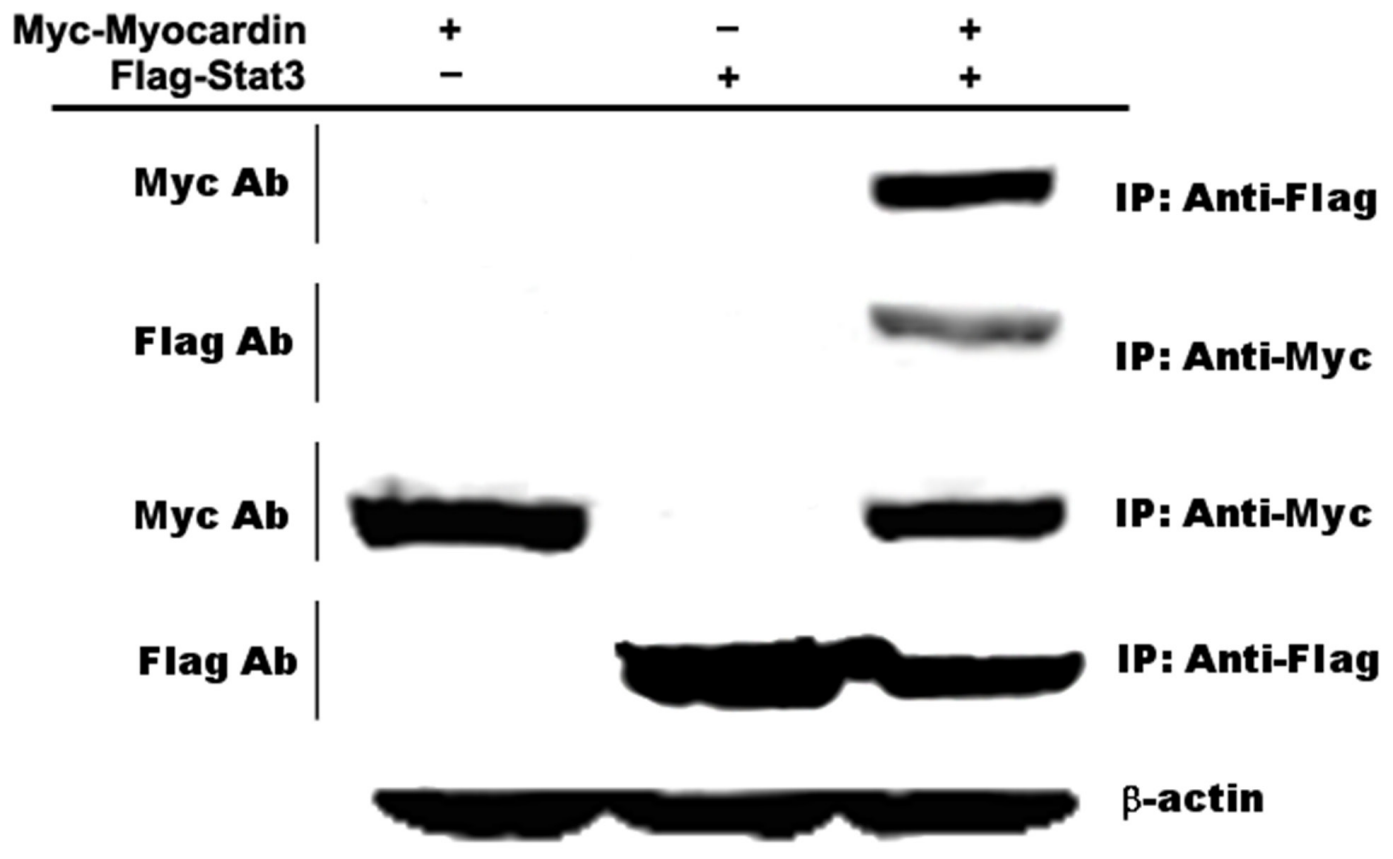
Myocardin and Stat3 interact *in vivo* Coimmunoprecipitation assays were used to identify the interaction of Myocardin and Stat3 after cotransfection of cardiomyocytes with myc-Myocardin and flag-Stat3 for 24 hours.

Myocardin, a coactivator of serum response factor (SRF) was reported to be a key regulator of cardiac and smooth muscle differentiation [17]. SRF as a direct regulator of Bcl-2 transcription as a novel mechanism is important for cell survival during embryonic development [20]. Stat3-mediated transcription of Bcl-2 and Mcl-1 prevent apoptosis in polyamine-depleted cells [35]. Since Myocardin and Stat3 can affect cells development and significantly inhibit staurosporine-induced cardiomyocyte apoptosis, we hypothesized that Myocardin and Stat3 have a direct physical interaction during cardiomyocyte apoptosis. Coimmunoprecipitation assays were used in cardiomyocytes to identify an interaction of Myocardin and Stat3 in the cellular environment. Our results demonstrate a direct interaction between Myocardin and Stat3 *in vivo* (Figure 4).

### Molecular mechanism of Myocardin and Stat3 anti-apoptosis marker gene regulation

**Figure 5:**
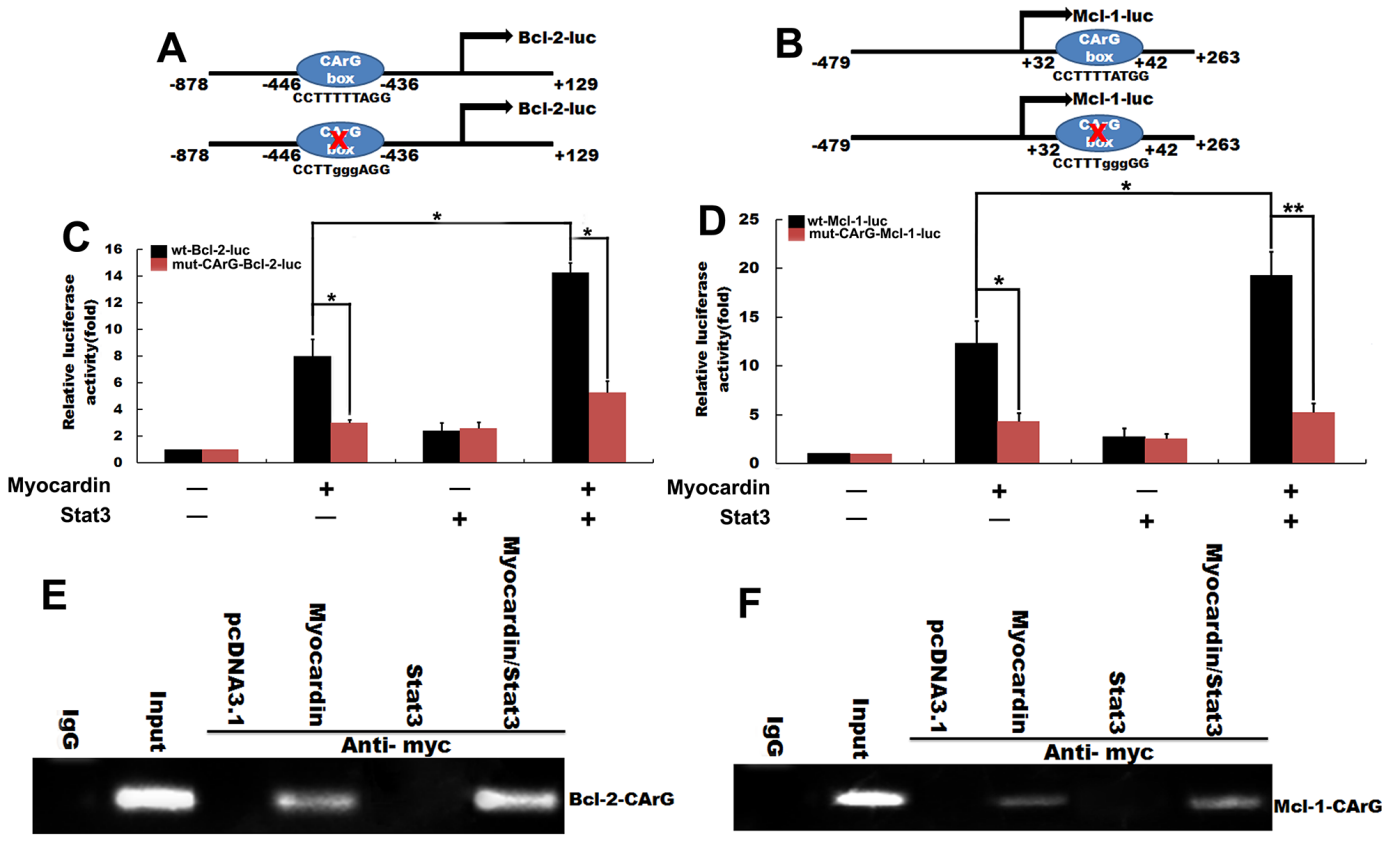
The molecular mechanism of Myocardin and Stat3 ranti-apoptosis marker gene regulation **(A)** Schematic of the -878 Bcl-2 promoter, containing the CArG box element, which was linked to a luciferase reporter and the -878 Bcl-2 promoter with a mutation in CArG box. **(B)** The postnatal rat cardiomyocytes were transfected with the wild-type -878 Bcl-2 promoter, or the -878 Bcl-2 promoter with a mutation in CArG box, and transfected with pcDNA3.1, Myocardin, Stat3, and Myocardin/Stat3 for 24 hours. Luciferase reporter assay was used to test the transactivity of Bcl-2. **(C)** Schematic of the -479 Mcl-1 promoter, containing CArG box elements and the -479 Mcl-1promoter with mutation CArG box. ^*^, *p*<0.05. n=6. **(D)** Postnatal rat cardiomyocytes were transfected with the wild-type -479 Mcl-1 promoter, or the -479 Mcl-1 promoter with a mutation in CArG box, and transfected with pcDNA3.1, Myocardin, Stat3, and Myocardin/Stat3 for 24 hours. Then the luciferase reporter assay was used to test the transactivity of Mcl-1. ^**^, *p*<0.01, ^*^, *p*<0.05. n=6. **(E and F)** The postnatal rat cardiomyocytes were transiently transfected with pcDNA3.1, Myocardin, Stat3, and Myocardin/Stat3 for 24 hours. ChIP assays were performed with primers for sequences associated with the genes for Bcl-2 and Mcl-1. Sheared DNA/protein complexes were immunoprecipitated by using an anti-myc Ab. Then, PCR was used to detect the endogenous CArG regions in immunoprecipitated chromatin fragments. The amount of DNA in each sample (input) is shown at the second land. Immunoprecipitations were performed with IgG as a negative control.

We constructed a luciferase promoter of Bcl-2 and Mcl-1 containing the site of CArG and measured the luciferase activity after transfection with the expression plasmid of Myocardin and Stat3 (Figure 5A and 5B) to determine whether Myocardin and Stat3 can promote the transcription of Bcl-2 and Mcl-1. Our data show that Stat3 can lightly enhance the Bcl-2 reporter gene expression, Bcl-2 reporter gene was markedly activated by Myocardin, and cotransfection of the two plasmids significantly improved the activity of the Bcl-2 promoter (Figure 5C). Similarly, cotransfection with Myocardin and Stat3 stimulated the transcriptional activity of the Mcl-1 promoter. These data show that Myocardin and Stat3 significantly enhanced the Bcl-2 and Mcl-1 reporter activities (Figure 5D).

Myocardin drives transcription by forming a stable complex with SRF bound to the CArG box commonly found in many smooth muscle and myofibroblast gene promoters. SRF binds directly to the murine Bcl-2 regulatory region *in vitro* and *in vivo* and activates Bcl-2 promoter-driven reporter gene transcription during early murine embryogenesis [20]. SRF binds to the cognate sites in the -107-bp region of Mcl-1 to increase the expression of Mcl-1through SRF-stimulated pathways [21]. To determine whether Myocardin/Stat3-mediated Bcl-2 and Mcl-1 promoter transactivation is dependent on the CArG box, we mutated the CArG box. As expected, CArG box mutation in the proximal promoter abolished Bcl-2 promoter activity (Figure 5C). Similarly, mutation of CArG box in the proximal promoter abolished Mcl-1 activity (Figure 5D).

ChIP assays further confirmed the impact of Myocardin/Stat3 on the Bcl-2 and Mcl-1 gene promoters. ChIP assays provided direct evidence for the involvement of Myocardin/Stat3 in the transcription of the endogenous Bcl-2 and Mcl-1 gene within the context of intact chromatin. Our results show that Myocardin and Myocardin/Stat3 bind the CArG box of the Bcl-2 promoter (Figure 5E) and the Mcl-1 promoter (Figure 5F). These results establish that the CArG box is necessary and sufficient for Myocardin mediated Bcl-2 and Mcl-1 promoter activity in cardiomyocytes.

## DISCUSSION

Apoptosis is associated with loss of cardiomyocytes following myocardial infarction, atherosclerotic plaque instability, and congestive heart failure [36]. Since cardiomyocyte loss is the most important determinant of patient morbidity and mortality, fully understanding the regulatory mechanisms of apoptotic signaling is crucial. Apoptosis can be initiated by caspase-dependent or -independent mechanisms [37], and Bcl-2 family members are key regulators of the apoptotic pathway: Bcl-2, Bcl-xl, or Mcl-1 inhibit caspase activation [38]. We revealed a new role for Myocardin and Stat3-regulated apoptotic and anti-apoptotic gene expression to regulate cardiomyocyte apoptosis. We conclude that Myocardin and Stat3 have a synergistic effect myocardial protection in cardiomyocyte apoptosis from our novel discovery that Myocardin and Stat3 protect cells from apoptotic cell death by regulating anti-apoptosis gene Bcl2 and Mcl-1 activity.

Stat3 signaling is an important molecular pathway that regulates cell renewal, differentiation, and apoptosis of various cell types [4–7]. Many studies have shown that Stat3 plays oncogenic roles by promoting the expression of cancer-associated genes such as cyclinD1, c-Myc, Cox-2, and Bcl-2 [39–42]. Stat3-mediated transcription of Bcl-2, Mcl-1, and c-IAP2 prevents tumor necrosis factor-α (TNF-α)-induced apoptosis in polyamine-depleted cells [35]. However, TNF-α induced cardiomyocyte apoptosis is mitigated by IL-10 treatment via the upregulation of Akt phosphorylation that further increases Stat3 phosphorylation [43]. Constitutive cardiomyocyte-restricted deletion of Stat3 has been found to result in increased apoptosis and increased susceptibility to doxorubicin-induced heart failure [13]. Our data showed that Stat3 significantly weakened the cardiomyocyte apoptosis rate induced by staurosporine and promoted the expression of the anti-apoptotic genes Bcl-2 and Mcl-1.

Myocardin is expressed specifically in cardiac and smooth muscle cells and potently activates their gene expression by associating with SRF bound to CArG boxes [17–19]. Previous studies have demonstrated that SRF promotes differentiating murine embryonic stem (ES) cell survival *in vitro* by binding to the Bcl-2 promoter *in vivo* and activating Bcl-2 transcription [20]. Our present study demonstrated that Myocardin also promotes cell survival by regulating the expression of anti-apoptotic genes. Firstly, we found that Myocardin inhibits staurosporine-induced cardiomyocyte apoptosis by TUNEL assay and Annexin V-FITC apoptosis detection assay. Cardiomyocyte cells transfected with Myocardin had reduced annexin-V expression on the cell surface as compared to control, which implicates Myocardin in cardiomyocyte apoptosis (Figure 1). The Bcl-2 family, consisting of both pro-apoptotic and anti-apoptotic proteins, is a critical checkpoint of the apoptosis pathway [44]. Mcl-1 has also been found to exhibit both similarities to and differences from Bcl-2 [45]. We further investigated the effect of Myocardin in regulating the expression of Bcl-2 and Mcl-1 in the presence of staurosporine. Our data showed that the expression of both Bcl-2 and Mcl-1 increased slightly after transfection with Myocardin. Based on these findings we concluded that Myocardin could weaken staurosporine-induced cardiomyocyte apoptosis and increase expression of anti-apoptotic Bcl-2 and Mcl-1. Importantly, cotransfected Myocardin and Stat3 to cardiomyocytes obviously reduced the number of apoptotic cells. Moreover, cotransfection of Myocardin and Stat3 synergistically increased the mRNA levels and protein levels of anti-apoptotic proteins Bcl-2 and Mcl-1 (Figure 2A and 2B). These observations support Myocardin and Stat3 mediated cardiomyocyte protection from apoptosis through enhancement of anti-apoptotic gene expression.

To clarify the regulatory mechanism we also identified the direct physical interaction of Myocardin and Stat3 by coimmunoprecipitation assays in cardiomyocytes. We found that transcription factors Myocardin and Stat3 interact directly *in vivo* (Figure 4). Given that the ratio of anti-apoptotic proteins such as Bcl-2 relative to pro-cell death proteins such as Bax determines the ultimate sensitivity of cells to various apoptotic stimuli [46], we calculated the ratio of Bcl-2 expression to Bax expression in the present study. The densitometric analysis revealed a high Bcl-2/Bax ratio in transfected Myocardin and Stat3 cardiomyocytes. From these findings, we conclude that Myocardin and Stat3 increase the expression of Bcl-2 while reducing the pro-apoptotic gene-Bax, which changes the ratio of Bcl-2/Bax to regulate the process of cardiomyocyte apoptosis.

Next, we constructed a luciferase promoter of Bcl-2 and Mcl-1 containing the site of CArG to study the mechanism of Myocardin and Stat3 anti-apoptosis marker gene regulation. Our data show that the cotransfection of Myocardin and Stat3 significantly enhance the transcriptional activity of Bcl-2 (Figure 2). Similarly, Myocardin and Stat3 significantly promote the transcriptional activity of Mcl-1. It has been demonstrated that SRF binds to the cognate sites in the -107-bp region of the Mcl-1 to increase the expression of Mcl-1through SRF-stimulated pathways [21]. The CArG box in the Bcl-2 promoter motif is necessary for activation of Bcl-2 transcription by SRF in ES cells *in vivo* [20]. We determined whether Myocardin/Stat3-mediated Bcl-2 and Mcl-1 promoter transcriptional activation depend on the CArG box. We found that mutation of CArG box in the proximal promoter abolished Myocardin/Stat3 stimulated Bcl-2 and Mcl-1 promoter activity *in vitro*. This observation confirmed our hypothesis that Myocardin/Stat3 promotion transactivity of Bcl-2 and Mcl-1 is dependent on CArG box. ChIP assays provide consistent and direct evidence of Myocardin/Stat3 involvement in endogenous Bcl-2 and Mcl-1 gene transcription within the context of the intact CArG box (Figure 5E and 5F).

In summary, the present study reveals a novel Myocardin/Stat3 anti-apoptotic gene signaling pathway that is operative during cardiomyocyte apoptosis and a molecular explanation for Myocardin/Stat3 inhibition of cardiomyocyte apoptosis and myocardial protection. Cardiomyocyte apoptosis is a crucial process in heart disease, including the progression from the initiation of hypertrophy (compensatory phase) to decompensation and transition to heart failure. Mechanistic insight into cardiomyocyte apoptosis regulation has led to novel therapeutic approaches. Using Myocardin/Stat3 (and analogs) to inhibit cardiomyocyte apoptosis holds promise as an effective therapeutic strategy for cardiovascular diseases.

## MATERIALS AND METHODS

### Plasmids, cell culture, cell treated, cell transfection

Myc-pcDNA3.1-Myocardin encoded the full-length mouse Myocardin, and flag-pcDNA3.1-Stat3 encoded the full-length mouse Stat3. Bcl-2 (-878 to +129) or Mcl-1 (-479 to +263) luciferase reporters were constructed in pGL3-basic plasmid (Promega).

The primers used to create Bcl-2-luc and Mcl-1-luc were as follows: Bcl-2: 5’ GGGTGGTACCAGGAGGGCTCTTTCTTTCTTCTTTT 3’ (sense) and 5’ GGCA AGCTTCCCATCAATCTTCAGCACTCTCCAG3’ (antisense); Mcl-1: 5’ CT CTCGAGAATGGTTCTTTAGGGTAGCACGTGG3’ (sense) and 5’ CAAAGCTT CCCCCCCACAGTAGAGGTTGAGTC3’ (antisense);

Mutations of the CArG sequences were performed with the QuickChange Site-directed Mutagenesis Kit (Stratagene) using the following mutagenesis primers:

Bcl-2 CArG mut: F: 5’-AACTTCGTAGCAGTCATCCTTgggAGGAAAAGAGGGAAAAAATAA-3’
Bcl-2 CArG mut: R: 5’-TTATTTTTTCCCTCTTTTCCTCCCAAGGATGACTGCTACGAAGTT-3’;
Mcl-1 CArG mut: F: 5’-AAGCTGCCGCCCCTTTCCCCTTTggg GGGAATACTTTTTTGGCGGC|GCGCG-3’;
Mcl-1 CArG mut: R: 5’-CGCGCGCCGCCAAAAAAGTATTCCCCCCAAAGGGGAAAGGGGCGGCAGCTT-3’;

Cardiomyocytes were isolated from d1-3 Sprague-Dawley rat pups as described previously [47–50], starved without serum for another 12 hours, and plated in growth medium without antibiotics. Cells were treated at 30-50% confluency with 1μM staurosporine (Sigma) for 30 min to induce cardiomyocyte apoptosis. Cardiomyocytes were transfected with plasmids pcDNA3.1, Myocardin, Stat3, and Myocardin/Stat3 with FuGENE^®^HD (Roche) according to manufacturer instructions. After transfection, the medium was removed and replaced with normal culture medium.

### RNA isolation and semiquantitative reverse-transcription polymerase chain reaction (RT-PCR)

Semiquantitative RT-PCR analysis was carried out as described previously [50]. Briefly, total RNA was isolated from cells using Trizol reagent (Invitrogen), the samples were reverse-transcribed by using M-MLV reverse transcriptase (Invitrogen) according to the manufacturer’s instructions. The PCR primer sequences are as follows:

GAPDH: F-ATTCAACGGCACAGTCAAGG, R-GCAGAAGGGGCGGAGATGA;
Bcl-2: F-CGGGAGAACAGGGTATGA, R-CAGGCTGGAAGGAGAAGAT;
Mcl-1: F-AAGAGGCTGGGATGGGTT, R-TTGGTGGCTGGAGGTTTT;
Bax: F-TGGTTGCCCTCTTCTAC, R-AGCCACCCTGGTCTTG;
Cytochrome c: F-GGCAAGCATAAGACTGGA, R-GGTCTGCCCTTTCTCCCT;
Apaf-1: F-CCTGAAGCCCAAGTGAGT, R-TGCCCTGATGACAAGTAAAG;
Caspase-9: F-CCCCACCCTCACTTTGCT, R-TAAGTGAGCCTGGTCCTCC;
Caspase-3: F-CTGGACTGCGGTATTGAG, R-GGGTGCGGTAGAGTAAGC;

Semiquantitative analysis of mRNA expression was performed with Biorad software. Glyceraldehyde-3-phosphate dehydrogenase (GAPDH) expression was used as an internal control.

### Protein isolation and western blotting

The procedure for Western Blotting has been described previously [51]. After treatment, cells were lysed in lysis buffer. Proteins were separated by SDS-PAGE, then transferred to an NC membrane (Pall) and blocked for 60 min at room temperature in 5% skim milk powder (wt/vol) and PBST (PBS + 0.05% Tween-20, vol/vol). After incubation with mouse polyclonal antibodies (goat anti-mouse Bcl-2 (Abcam), goat anti-mouse Bax (Abcam), goat anti- mouse Mcl-1 (Abcam), goat anti- rabbit Apaf-1 (Cell Signaling Technology), goat anti- rabbit Cleaved-caspase-9 (Cell Signaling Technology), and goat anti- rabbit Cleaved-caspase-3 (Cell Signaling Technology), the appropriate secondary antibodies (IRDye™ 800-goat anti-mouse, IRDye™ 800-goat anti-rabbit, IRDye™ 680-goat anti-mouse, IRDye™ 680- goat anti-rabbit, Li-COR) were added for incubation. Specific proteins were visualized with an Odyssey Infrared Imaging System. Mouse anti β-actin (Santa Cruz) expression was used as an internal control.

### Apoptosis assay

Apoptosis was determined through externalization of plasma membrane phosphatidylserine (PS) and TUNEL assay analysis

### Annexin V-FITC apoptosis detection assay

An increase in the plasma membrane PS externalization occurs early in apoptosis and can be detected by Annexin V staining. Cardiomyocytes were treated with staurosporine 30 min as described above, then transfected with plasmids pcDNA3.1, Myocardin, Stat3, and Myocardin/Stat3 for 24 hours. Cardiomyocytes were isolated, stained with annexin V-FITC (BioVision Research Products, Mountain View, CA), and analyzed using FACS (BD Accuri C6, USA) for fluorescence of annexin V-positive cells.

### TUNEL assay for cardiomyocyte apoptosis

The terminal deoxynucleotidyl transferase-mediated dUTP nick-end labeling (TUNEL) assay was used to monitor the extent of DNA fragmentation as a measure of apoptosis in paraffin-embedded sections. Cardiomyocytes were fixed with formaldehyde. Immunohistochemical detection of apoptotic cells was carried out with a TUNEL reaction by using DeadEnd™ Fluorometric TUNEL System (Promega). The cells were washed 3 times with PBS, blocked with 10% normal goat serum in PBS, and incubated with DAPI (4’,6’-diamidino-2-phenylindole) to stain the nuclei. The samples were evaluated under inverted confocal laser microscope (Olympus, Japan). For the quantitative purpose, TUNEL-positive cardiomyocytes were counted via microscope.

### Coimmunoprecipitation

Plasmid-based expression vectors encoding myc-tagged myocardin and flag-tagged Stat3 were cotransfected into cardiomyocytes. Lysates were collected 48 hours post-transfection. Then myc antibody (Santa Cruz) was used to precipitate myc-myocardin, and flag antibody (Santa Cruz) was used to precipitate flag-Stat3. The resulting mixture was washed, separated by SDS-PAGE, and transferred to an NC membrane (Pall). Samples were probed with myc antibody to visualize flag-epitope Stat3, stripped, and probed with flag antibody to visualize myc-epitope Myocardin.

### ChIP assay

ChIP analysis was performed using a commercially available kit (Enzymatic Chromatin IP (Magnetic Beads), Cell Signaling Technology, USA) on cardiomyocytes transfected with Myocardin, Stat3, and Myocardin/Stat3 for 24 hours. DNA-bound proteins were cross-linked using formaldehyde at a final concentration of 1% for 20 min at room temperature. Protein-DNA complexes were immunoprecipitated using primary antibodies for myc antibody (Santa Cruz). Myocardin and Mcl-1 promoter complexes, Stat3 and Mcl-1 promoter complexes, and Myocardin, Stat3 and Mcl-1 promoter complexes were measured by PCR. The primers used for the amplification of the human promoter region between -550 to -311bp were: Bcl-2-CArG box forward 5’GCAGGACCAGGAGGAGGA 3’, reverse 5’ GATAAATGAAGGCAGGACGC 3’; human Mcl-1 promoter region between -113 to +140bp were: Mcl-1-CArG box forward 5’ CACTCAGAGCCTCCGAAGACC 3’, and reverse 5’ GAAGTGAGAAGTGGCGAGCAG 3’. The samples were separated by electrophoresis in 2% agarose gel and visualized by ethidium bromide staining.

### Immunocytochemistry assay

Immunofluorescence assays were performed as described previously [52]. After adding the primary antibodies (mouse anti-Bcl-2(Abcam), mouse anti-Mcl-1(Abcam)), we added appropriate secondary antibodies (FITC-goat anti-mouse IgG Santa Cruz) to the samples. DAPI was used to stain nuclei. The samples were then evaluated under an inverted laser scanning confocal microscope (OLYMPUS-FV-1000, Japan).

### Luciferase assay

Luciferase assays were performed as described previously [51]. Cardiomyocytes were seeded onto 24-well plates (Corning). Transfections were performed with FuGENE^®^HD (Roche) according to manufacturer protocol. The total amount of DNA per well was kept constant by adding the corresponding amount of expression vector without a cDNA insert. After transfection for 24 hours, luciferase activity was measured with a luciferase reporter assay system (Promega) on a Synergy™ 4 Hybrid Microplate Reader (Biotek). Transfection efficiencies were normalized to the total protein concentration of each luciferase assay preparation. All experiments were performed in triplicate with different preparations of plasmids and primary cells. Data of representative experiments are presented as the mean ± standard deviation of triplicates.

### Statistical analysis

Data are presented as mean ± SE and accompanied by the number of experiments performed independently, and analyzed by *t-test*. Differences with *P*<*0.05* were considered statistically significant.

## Abbreviations

Stat3: Signal transducer and activator of transcription 3
Mcl-1: myeloid cell leukemia-1
Apaf-1: apoptotic protease activating factor-1
TNF-α: tumor necrosis factor-α
ES: embryonic stem
GAPDH: Glyceraldehyde-3-phosphate dehydrogenase
PS: phosphatidylserine
TUNEL: The terminal deoxynucleotidyl transferase-mediated dUTP nick-end labeling
DAPI: 4’,6’-diamidino-2-phenylindole
